# The Role of Electroconvulsive Therapy in the Treatment of Catatonia Associated With Lewy Body Dementia: A Case Report

**DOI:** 10.7759/cureus.52500

**Published:** 2024-01-18

**Authors:** Javeria Sahib Din, Thomas Boes, Ernesto Navarro Garcia, Hiba Al-Rubaye

**Affiliations:** 1 Psychiatry, Manhattan Psychiatric Center, New York, USA; 2 Neuropsychiatry, Manhattan Psychiatric Center, New York, USA; 3 Nanotechnology, University of Central Florida, Orlando, USA; 4 Neuroscience, St. George's University School of Medicine, St. George's, GRD

**Keywords:** electro-convulsive therapy, dementia with lewy bodies, bush francis catatonia rating scale, benzodiazepines, catatonia

## Abstract

Catatonia is a complex amalgamation of neuropsychiatric symptoms that can manifest in both psychiatric and neurological conditions. The treatment of catatonia related to psychiatric illnesses is well documented as it typically responds to benzodiazepines and electroconvulsive therapy (ECT). However, the treatment of catatonia related to neurological disorders has shown to be more difficult, particularly when associated with Lewy Body Dementia (LBD). Here, we present the case of a 78-year-old woman with LBD, Bipolar I, depressive type, who successfully underwent twelve ECT sessions to treat catatonia refractory to benzodiazepine therapy. The effectiveness of the treatment was measured using the Bush-Françis Catatonia Scale (BFCS) to measure her catatonic symptoms as she progressed through the therapy. This report highlights the importance of considering ECT as a leading therapeutic approach in this particular subset of patients who do not respond adequately to pharmaceutical therapy and medical titrations.

## Introduction

Lewy body dementia (LBD) is known for encompassing two different clinical conditions: dementia and parkinsonism. This disease is characterized by dementia, which includes memory problems and altered cognition, which may include personality changes such as paranoia, irritability, delusions, anxiety, and apathy among others. Other significant symptoms include visual hallucinations and motor symptoms that mimic parkinsonism with rigidity, tremors, reduced facial expressions, and posture instability [1]. Although both dementia and parkinsonism can occur in the same individual over time, the diagnosis is primarily based on the onset and time of certain symptoms, particularly the onset of dementia, versus the onset of motor symptoms. Current diagnostic criteria follow research rules, which differentiate the diseases based on a one-year period difference between the onset of dementia symptoms and motor symptoms. Specifically, if the onset of dementia symptoms begins one year prior to the motor symptoms, the diagnosis is consistent with LBD [2,3]. Current treatment guidelines for LBD focus on multifaceted therapy, as no cure is currently available. Symptomatic treatment for cognitive symptoms with cholinesterase inhibitors, neuropsychiatric symptoms with antipsychotics, and motor symptoms with levodopa are among the main targets in treatment [4].

Among the plethora of symptoms that affect individuals with the illness, catatonia is one of the most salient as it can make diagnosis inaccurate, treatment difficult, and on its own can be potentially lethal [5]. Catatonia has been previously and extensively discussed. Briefly, catatonia involves four major areas: pure motor (e.g., posturing, rigor, immobility), disturbances of will (e.g., negativism, automatic obedience, agitation), unsuppressed complex motor activity (e.g., stereotypy, echolalia, echopraxia, mutism, mannerisms, grasp reflex), and autonomic components (e.g., tachycardia, hypertension, fever) [6-8]. 

Current data on the pathophysiology of catatonia suggests a bevy of mechanisms involved. Among the leading suggested mechanisms, hyperperfusion of the supplementary motor area (SMA) and ventral premotor cortex (vPMC) may also drive the basal ganglia’s inhibitory effects via the direct pathway to the substantia nigra, decreased activation of the primary motor cortex, improper connections between the thalamus and motor cortices as related to the direct pathway, and a reduction of GABA A receptor density in the primary motor cortex have been attributed [9-15]. Altogether, it is possible that these underlying mechanisms partake in the complex presentation of catatonia. The treatment of catatonia primarily involves benzodiazepines, particularly lorazepam. Other agents include N-methyl-D-aspartate (NMDA) antagonists such as amantadine and memantine, sedative-hypnotics like zolpidem, mood stabilizers/antipsychotics such as carbamazepine or valproate, and electroconvulsive therapy. Studies have shown variable responses to benzodiazepines; some cases show no significant clinical improvement, while others have achieved full remission, ECT has similar results. Antipsychotics such as olanzapine, quetiapine, and risperidone have shown poor mixed responses [16]. It is important to mention that these studies focused on the use of pharmacotherapy and ECT predominantly in catatonia associated with psychosis and mood disorders. The treatment of catatonia associated with dementia, such as LBD is scarce but has been reported to help in some cases [17].

## Case presentation

The patient is a 78-year-old French-American female, with a past medical history of essential hypertension, intrinsic eczema, mixed hyperlipidemia, slow transit constipation, and active psychiatric diagnosis of bipolar I, unspecified catatonia. The patient initially presented with symptoms of depression and suicidal thoughts lasting several weeks before admission to the psychiatric institution. During the initial examination, the patient was dysphoric and intermittently tearful, with persistent passive suicidal ideation, somatic preoccupations, and anxiety. The patient was also noted to have a tangential thought process, occasional irritability, and expressed nihilistic delusions of being "dead-brained" or a "dead person". The patient also attempted suicide while being in the unit. A few months later, the patient continued to decline and demonstrated a slow shuffling gait, cogwheel rigidity on upper extremities, and difficulty eating. The diagnosis of Lewy Body Dementia (LBD) was based on the clinical presentation of dementia symptoms followed by parkinsonism more than a year after the onset of cognitive decline. Furthermore, her cognition was fluctuating on a daily basis, a key sign of LBD [18]. Additionally, the patient showed extreme sensitivity to antipsychotic medications with marked extrapyramidal symptoms to low-dose typical and atypical neuroleptics. Sleep was disturbed early in her admission with witnessed thrashing in her sleep. Psychiatrically, she developed Capgras delusions, typical of neurodegenerative disease. At this time, no radiological imaging, laboratory studies, or toxicology studies were pursued given her clinical presentation.

Symptoms of catatonia began shortly after the diagnosis of LBD with mutism, low-grunting sounds, purposeless movements (e.g., walking backward despite staff intervention), repetitive hand rubbing, facial grimacing, motor hesitancy, and poor oral intake. Notably, she needed help to maintain her activities of daily living (ADLs). At this time, the patient was started titrated to lorazepam 4 mg orally (PO) total daily dose (TDD). During this titration, the patient exhibited moments of lucidity, increased verbal communication, and became re-directable with less anxiety. However, her doses of lorazepam fluctuated due to episodes of increasingly unsteady gait, placing the patient at high fall risk. Initially, the patient improved, became less agitated and increasingly verbal, required less assistance to tend to her ADLs and increased independence with other activities. However, psychotic symptoms worsened as the patient began to express nihilist delusions of “being dead” and “disappearing” with increased paranoia and belief the unit staff and her providers were imposters. Due to the patient's prior poor response to quetiapine, olanzapine, and clozapine, as well as intolerance of chlorpromazine, pimavanserin was initiated (34 mg PO daily, for psychosis with parkinsonism). Psychotic symptoms improved slightly; however, her concentration, alertness, and attention fluctuated from day to day and within the same day consistent with LBD. Rivastigmine was added to address cognitive fluctuations and behavioral dysregulation with partial benefit. Sinemet® (25/100 mg twice a day (BID))was tried for parkinsonism, but the patient showed paranoid delusions and agitation, prompting a trial of amantadine (100 mg PO daily) to help both parkinsonism and catatonia. She responded well initially with reduced parkinsonism and decreased Bush-Françis Catatonia Scale (BFCS) score, but then as the dose was increased, she expressed suicidal ideations (SI). Amantadine was stopped and a repeat trial of Sinemet® (25/100 mg BID) was begun for rigidity and akinesia but discontinued due to increased catatonic rigidity, mutism, verbigeration and finger play. Amantadine 100 mg PO daily was resumed. The patient was continuously monitored on a daily basis to assess if any changes occurred in personality, thoughts, SI, and movement that could've been attributed to the pharmaceutical changes.

Intermittent signs of catatonia began to reappear, patient demonstrated non-verbal behavior, increased grasp, constant lip pursing, occasional finger play, rigidity, and mitgehen, Bush-Françis score at this time was 1-14 8, 1-23, 13. This intermittent pattern of catatonic-like behaviors worsened as the patient began to exhibit constant finger play, mutism, severe negativism, repetitive lip pursing, grimacing, vocalization “pee pee poo poo”, and fixed downward gaze, Bush-Françis score increased to 27 as shown in Table 1.

**Table 1:** Pertinent scores in Bush-Françis Catatonia Scale pre-ECT treatment Scores 2 and 3 represent the values assigned to the category towards the total BFCS score based on existing criteria. The usual scoring criteria for the Bush-Françis Catatonia Scale (BFCS) implements values ranging from 0 (absent) to 4 (constant/present/severe). Score values 2 and 3 represent the degree to which the symptoms are present during examination (1 being minimal, and 3 being moderately present). The total score is 27. ECT: Electroconvulsive therapy Table created by authors ENG and HA.

Bush - Françis Catatonia Scale pre-ECT		
Category	Score	Associated symptoms
Immobility and rigidity	3	Sitting in a wheelchair with moderate rigidity.
Stereotype	3	Constant fingerplay.
Staring	2	Fixed gaze down, non-reactive.
Mutism	2	Occasional vocalization doesn’t follow commands.
Grimacing	3	Repetitive pursing of the lips.
Withdrawal	2	Refused food and drinks when offered on multiple occasions.
Negativism	3	As defined.
Grasp Reflex	3	Positive, as defined.
Mitgehen	3	As defined.
Gegenhalten	3	As defined.

At this time, the alternative therapies were sorted and explored. The addition of ECT was offered, and a court order was obtained. ECT therapy was begun using a bi-temporal brief pulse approach in a bi-weekly fashion with adequate seizures achieved. ECT was done using a Thymatron® IV machine (Somatics, LLC, USA). After a period of three ECT sessions, marked improvement was seen in motor activity, decreased bradykinesia, improved orientation, and complete resolution of catatonia. Sessions were changed from twice per week to a weekly basis to minimize the risk of ECT-related delirium and cognitive decline.

After six sessions, the BFCS score was 5, with pertinent scoring criteria shown in Table 2. After the completion of 12 sessions, the BFCS score was reduced to zero and the patient showed improvement in ADLs with less assistance necessary. The patient became increasingly verbal and was able to communicate concerns within reason; her motor movement became less rigid and less repetitive, and the patient became more capable of performing ADLs independently and was able to communicate with staff with more coherence. Catatonia is a complex phenomenon that is not thoroughly understood, in this case, the presence of other conditions such as bipolar I and parkinsonism could play a role in the success of ECT. However, it is worth noting that this patient was on Sinemet®, amantadine, pimavanserin, and sertraline prior to the ECT treatment, with no clinical improvement in her catatonic symptoms. At this time, the patient is still in the unit while being closely monitored for any changes. Current follow-up plans include continuing ECT therapy in attempts to reduce BFCS to zero; if achieved, ECT will be discontinued, and diagnostic imaging will be pursued via a DAT (Dopamine Active Transporter) scan.

**Table 2:** Pertinent scores in Bush-Françis Catatonia Scale post-ECT treatment in 6 sessions Scores 1 and 3 represent the values assigned to the category towards the total BFCS score based on existing criteria.  The usual scoring criteria for Bush-Françis Catatonia Scale (BFCS) implements values ranging from 0 (absent) to 4 (constant/present/severe). Score values 1 and 3 represent the degree to which the symptoms are present during the time of examination (1 being minimal, and 3 being moderately present). The total score is 5. Table created by authors ENG and HA.

Bush - Françis Catatonia Scale post-ECT		
Category	Score	Associated symptoms
Mutism	1	Verbally unresponsive, incomprehensible whisper.
Staring	1	Poor eye contact, repeatedly gaze less than 20 seconds between shifting of attention and decreased blinking.
Mitgehen	3	As defined.

## Discussion

Typically, the use of ECT is reserved for refractory depression or psychiatric conditions in which life-threatening symptoms such as suicidality or severe psychosis are noted. It benefits from having no absolute contraindications, but caution must be applied in patients with recent myocardial infarction, severe hypertension, and increased intracerebral pressure. Although the use of ECT is well reported for cases of schizophrenia, depression, and parkinsonism, as well as specific cases of catatonia [19-22], the use of ECT in dementia-related disorders is not well established. Furthermore, there is a negative stigma surrounding its use due to its poor portrayal in movies as barbaric and ancient, its fear of electricity, and its fear of unwanted memory loss as a potential side effect [23]. Some attempts at consolidating data have shown its potential use for dementia and its associated symptoms, such as agitation, and psychosis [24]. The standard treatment of catatonia is split into pharmacology and ECT [16]. When the catatonia is refractory to benzodiazepines, ECT has shown promising results in psychiatric cases.

In this case, the patient underwent titration of antipsychotic medications, addition of benzodiazepines, and other Parkinsonian drugs. The antipsychotic drugs worsened the extrapyramidal symptoms, and the use of benzodiazepines proved ineffective as her risk for falls increased due to sedative effects. During this period, catatonia remained refractory, and other symptoms were exacerbated during medical titrations. The decision to pursue ECT was made after several trials of pharmacotherapy and after weighing the risks and benefits. In this case, the court order was necessary due to the patient’s inability to understand the need for non-pharmacological therapy. Additionally, this patient had access to a mental health counselor who was legally appointed to defend the patient’s best interest, thus, a formal hearing was needed to ethically and reasonably obtain permission to pursue ECT. Bitemporal brief pulse ECT was started on a bi-weekly basis with adequate seizures achieved. After six sessions, ECT showed vast improvement in catatonia, consistent with improved scores on the Bush-Françis Catatonia Scale seen in Table 1.

Additionally, the Parkinsonian symptoms vastly improved as a secondary benefit of the treatment, as expected based on previous data [22]. Bi-weekly sessions were tapered to weekly sessions to diminish the potential side effects given the patient’s underlying dementia. Altogether, ECT should be considered in all refractory catatonia after appropriate pharmacological therapy, regardless of possible etiology.

Limitations

Current limitations for this case report include the presentation of several neuropsychiatric symptoms and comorbid diagnoses which may alter the efficacy of ECT. Namely, the patient presents with a diagnosis of bipolar I, parkinsonism, and LBD. Another factor to consider is the need for high-dose lorazepam to achieve clinical improvement, thus placing the patient at greater fall risk due to illness, medication, and age. Furthermore, the patient continuously needs court-ordered treatment due to her inability to comprehend her current medical situation which may delay the appropriate time for treatment, and the choice of treatment itself due to waiting times for court hearings to take place.

## Conclusions

The case of a 78-year-old woman with LBD and refractory catatonia demonstrated the limited efficacy of conventional treatment for catatonia associated with dementia. The patient’s journey through various pharmacotherapies revealed minimal success, leading to the decision to introduce ECT. The subsequent sessions demonstrated significant improvement in catatonic symptoms, as reflected in the Bush-Françis Catatonia Scale scores across 12 sessions. The secondary benefit of improved Parkinsonian symptoms highlights the duality of this treatment in dementia-related disorders, particularly LBD. This case advocates for the consideration of ECT in refractory catatonia associated with LBD, particularly when conventional pharmacotherapies prove ineffective.

## References

[1] Haider A, Spurling BC, Sánchez-Manso JC (2023). Lewy Body Dementia. https://www.ncbi.nlm.nih.gov/books/NBK482441/.

[2] Yamada M, Komatsu J, Nakamura K, Sakai K, Samuraki-Yokohama M, Nakajima K, Yoshita M (2020). Diagnostic criteria for dementia with Lewy bodies: updates and future directions. J Mov Disord.

[3] Menšíková K, Matěj R, Colosimo C (2022). Lewy body disease or diseases with Lewy bodies?. NPJ Parkinsons Dis.

[4] McKeith IG, Boeve BF, Dickson DW (2017). Diagnosis and management of dementia with Lewy bodies: Fourth Consensus Report of the DLB Consortium. Neurology.

[5] Burrow JP, Spurling BC, Marwaha R (2023). Catatonia. https://www.ncbi.nlm.nih.gov/books/NBK430842/.

[6] Bush G, Fink M, Petrides G, Dowling F, Francis A (1996). Catatonia. I. Rating scale and standardized examination. Acta Psychiatr Scand.

[7] Francis A (2010). Catatonia: diagnosis, classification, and treatment. Curr Psychiatry Rep.

[8] Dhossche DM (2014). Decalogue of catatonia in autism spectrum disorders. Front Psychiatry.

[9] Walther S, Strik W (2016). Catatonia. CNS Spectr.

[10] Northoff G, Braus DF, Sartorius A (1999). Reduced activation and altered laterality in two neuroleptic-naive catatonic patients during a motor task in functional MRI. Psychol Med.

[11] Payoux P, Boulanouar K, Sarramon C (2004). Cortical motor activation in akinetic schizophrenic patients: a pilot functional MRI study. Mov Disord.

[12] Walther S, Schäppi L, Federspiel A, Bohlhalter S, Wiest R, Strik W, Stegmayer K (2017). Resting-state hyperperfusion of the supplementary motor area in catatonia. Schizophr Bull.

[13] Mehta UM, Basavaraju R, Thirthalli J (2013). Mirror neuron disinhibition may be linked with catatonic echo-phenomena: a single case TMS study. Brain Stimul.

[14] Walther S, Stegmayer K, Federspiel A, Bohlhalter S, Wiest R, Viher PV (2017). Aberrant hyperconnectivity in the motor system at rest is linked to motor abnormalities in schizophrenia spectrum disorders. Schizophr Bull.

[15] Guzman CS, Myung VH, Wang YP (2008). Treatment of periodic catatonia with atypical antipsychotic, olanzapine. Psychiatry Clin Neurosci.

[16] Pelzer AC, van der Heijden FM, den Boer E (2018). Systematic review of catatonia treatment. Neuropsychiatr Dis Treat.

[17] Saito Y, Noto K, Kobayashi R, Suzuki A, Morioka D, Hayashi H, Otani K (2021). Catatonia as the initial manifestation of dementia with Lewy bodies. Am J Case Rep.

[18] Matar E, Shine JM, Halliday GM, Lewis SJ (2020). Cognitive fluctuations in Lewy body dementia: towards a pathophysiological framework. Brain.

[19] Grover S, Sahoo S, Rabha A, Koirala R (2019). ECT in schizophrenia: a review of the evidence. Acta Neuropsychiatr.

[20] Pagnin D, de Queiroz V, Pini S, Cassano GB (2004). Efficacy of ECT in depression: a meta-analytic review. J ECT.

[21] Vaquerizo-Serrano J, Salazar De Pablo G, Singh J, Santosh P (2021). Catatonia in autism spectrum disorders: a systematic review and meta-analysis. Eur Psychiatry.

[22] Takamiya A, Seki M, Kudo S, Yoshizaki T, Nakahara J, Mimura M, Kishimoto T (2021). Electroconvulsive therapy for Parkinson's disease: a systematic review and meta-analysis. Mov Disord.

[23] Sadeghian E, Rostami P, Shamsaei F, Tapak L (2019). The effect of counseling on stigma in psychiatric patients receiving electroconvulsive therapy: a clinical trial study. Neuropsychiatr Dis Treat.

[24] Williams D, Campbell K (2019). Electroconvulsive Therapy for the Treatment of the Behavioural and Psychological Symptoms of Dementia: A Review of Clinical Effectiveness and Guidelines. https://www.ncbi.nlm.nih.gov/books/NBK546016/.

